# Research Progress on Natural Polysaccharide Hydrogels in the Diagnosis and Treatment of Colorectal Cancer

**DOI:** 10.3390/gels12070590

**Published:** 2026-07-02

**Authors:** Hui Li, Jiafei Long, Songqiao Zha, Shengyi Zhuang, Mingqiu Liu, Yi Liu, Sanhua Li, Yanlei Guo, Gang Wang

**Affiliations:** 1 School of Pharmacy, Zunyi Medical University, Zunyi 563000, China; 18311889361@163.com (H.L.); ljf13639222319@163.com (J.L.); zhasongqiao_z@163.com (S.Z.); zhuangsy2142@163.com (S.Z.); 15185557954@163.com (M.L.); 13908563115@163.com (Y.L.); 2 Key Laboratory of Pharmaceutical Research for Tumor Prevention and Treatment, Department of Education of Guizhou Province, Zunyi 563000, China; zyzmclsh@163.com; 3 Chongqing Academy of Chinese Materia Medica, No. 34 Nanshan Road, Nan’an District, Chongqing 400065, China; 4 Guizhou Key Laboratory of Modern Traditional Chinese Medicine Creation, Zunyi 563000, China

**Keywords:** natural polysaccharides, hydrogels, CRC, classification, response type

## Abstract

Colorectal Cancer (CRC) is a prevalent global malignant tumor, and conventional therapies and drugs for CRC are limited by poor targeting and severe toxic side effects. Existing reviews on hydrogel-based CRC treatments mainly focus on synthetic materials or single-responsive systems concerning drug loading and local delivery. Natural polysaccharides possess inherent anti-inflammatory, antioxidant and antitumor activities, and polysaccharide hydrogels (PSHs) prepared from them exhibit favorable biocompatibility, tunable structures and potential targeting capability, which can synergistically enhance the efficacy of loaded drugs and thus become a research hotspot. This article summarizes the pathogenesis and conventional treatments of CRC, introduces monocomponent and composite PSHs as well as physical and chemical crosslinking methods, and emphasizes their tumor microenvironment (TME)-responsive mechanisms, combined drug effects and clinical applications. It also analyzes the challenges in safety evaluation and practical application, and summarizes recent advances in the use of artificial intelligence (AI) for PSHs design, regulation, performance prediction and implementation. This paper serves as a reference for follow-up research and clinical translation of hydrogels prepared from natural polysaccharides for the treatment of CRC.

## 1. Introduction

CRC is a malignant tumor originating from the epithelial cells of the colon or rectum. The pathogenesis of CRC follows the “adenoma–carcinoma sequence,” wherein normal mucosal tissues, under the influence of genetic and metabolic abnormalities, gradually undergo malignant transformation through the adenomatous polyp stage [1]. The risk factors of CRC are categorized into environmental and genetic factors: environmental factors include alcohol consumption, smoking, obesity, and gut microbiota dysbiosis, while genetic factors involve age, family history, and inflammatory bowel disease [2]. Regarding prevalence, developed countries in Europe and the United States have a high baseline incidence, whereas the incidence rate in developing countries is increasing rapidly. Globally, the number of early-onset CRC cases (in individuals under 50 years of age) is on the rise year by year [3]. As the third most common malignant tumor worldwide, CRC accounted for 2 million new cases globally in 2020. It is projected that by 2040, the number of new cases will increase to 3.2 million, with 1.6 million associated deaths, and the incidence rate in males is 44% higher than that in females [4]. CRC poses significant hazards: in the advanced stage, it is prone to drug resistance, recurrence, and distant metastasis and is accompanied by a low five-year survival rate. Postoperative anastomotic leakage is a common complication, and combined with the limitations of traditional therapeutic approaches, the overall diagnosis and treatment of CRC still face numerous challenges [5].

CRC has evolved from traditional therapeutic approaches to the era of precision medicine. Early-stage interventions, including surgery, radiotherapy, and chemotherapy, are invasive and associated with significant adverse effects. Although therapies in the precision medicine era—such as immunotherapy, targeted therapy, and photodynamic therapy [6]—exhibit prominent advantages of high targeting specificity and non-invasiveness, they still encounter challenges that include insufficient reagent selectivity, poor drug solubility, and difficulty in controlling adverse reactions [7]. The emergence of nanomedicines provides a novel strategy for overcoming tumor drug resistance. Inorganic, lipid-based, and polymeric nanoparticles can load antitumor agents to achieve targeted delivery and multimodal synergistic therapy; however, they still suffer from limitations such as rapid drug burst release, inadequate targeting ability, rapid in vivo clearance, and poor degradability [8]. Against this background, the integration of hydrogels and nanotechnology has become a research focus. Hydrogels can form in situ to construct drug sustained-release reservoirs, disperse nanoparticles to avoid agglomeration, and possess both multifunctional properties and drug protection effects [9]. Among these, PSHs have become the preferred carrier in this field, as the gastrointestinal tract lacks carbohydrate enzymes capable of digesting most natural polysaccharides, enabling targeted drug delivery to the colon and realizing specific release [10].

Natural PSHs are three-dimensional network materials formed by physical or chemical cross-linking of natural polysaccharides, including sodium alginate, carboxymethyl chitosan, and starch. These materials can absorb and retain large quantities of water without dissolution, and they possess excellent biocompatibility, biodegradability, high water absorption capacity, swelling properties, stimulus responsiveness, and environmental benignity [11]. Furthermore, they exhibit multifunctional characteristics such as self-healing, injectability, antibacterial activity, and antioxidant capacity, which render them ideal biomedical delivery carriers. In the treatment of CRC, PSHs demonstrate remarkable advantages: (A) targeted and intelligent responsiveness; (B) excellent biocompatibility and degradability, which mitigate immune reactions; (C) high drug-loading capacity coupled with sustained drug release; (D) enhanced drug solubility and stability, thereby improving the bioavailability of hydrophobic drugs in CRC treatment; and (E) stimulus responsiveness that enables controlled release triggered by the TME. In contrast, traditional drug delivery systems for CRC are plagued by limitations, including poor targeting, significant systemic adverse effects, and vulnerability to degradation by gastric acid or rapid clearance from the bloodstream, which substantially compromise delivery efficiency [12].

Numerous reviews have summarized the research progress of in situ hydrogels for CRC treatment. Some discussed material design, combined therapies and clinical translation of synthetic and composite hydrogels, while others overviewed hydrogel-based multimodal therapy, responsive mechanisms and clinical research. However, most existing reviews focus on common or synthetic polymer hydrogels or only elaborate on a single polysaccharide or therapeutic strategy. Systematic reviews targeting natural PSHs are scarce, and in-depth discussions on their biosafety, advanced technology integration and diagnosis-treatment combination are also lacking [7]. This paper therefore conducts a targeted review on natural PSHs for CRC therapy, covering CRC pathological features, PSH classification, clinical applications and biosafety evaluation, and explores their innovative development with AI integration. It fills the gaps in previous studies and provides theoretical support for the optimization, mechanistic research and clinical translation of natural PSHs in anti-CRC treatment.

## 2. An Overview of CRC

### 2.1. Pathological Mechanisms of CRC

**Figure 1:**
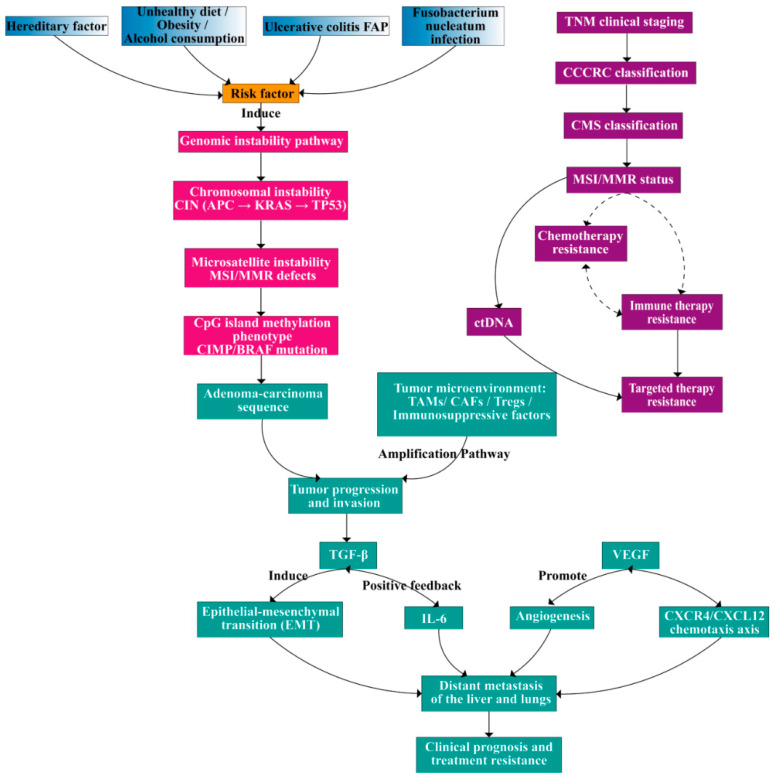
Schematic overview of the multi-step pathological progression and molecular regulatory mechanisms of CRC.

The occurrence of CRC is centered around the “adenoma–carcinoma sequence” (as shown in Figure 1), driven by abnormal molecular pathways, genetics, environment, and the intestinal microecology [13]. Among them, chromosome instability (CIN), microsatellite instability (MSI), and the CpG island methylation phenotype (CIMP) are the three core pathways that dominate carcinogenesis. Family genetics, ulcerative colitis, poor diet, and Fusobacterium nucleatum infection all increase the risk of disease. The invasion and metastasis of CRC depend on the epithelial–mesenchymal transition (EMT) of tumor cells [14], angiogenesis formation, and regulation of the TME. The VEGF pathway and the CXCR4/CXCL12 axis respectively promote angiogenesis and tumor cell metastasis to the liver and lungs. Immune-suppressive cells in the TME and different molecular subtypes also affect the invasion process. At the same time, tumor cells undergo metabolic reprogramming to supply energy and form treatment resistance. The prognosis of CRC is mainly affected by TNM staging, molecular subtypes, etc. [15]. Early-stage patients have a better prognosis, while patients with CMS4 subtype and stage IV combined with BRAF V600E mutation have a poorer prognosis. Treatment resistance involves multiple dimensions of chemotherapy, targeted therapy, and immunotherapy and is closely related to related gene mutations, immune suppression, and abnormal pathways. In summary, early diagnosis, precise classification, and targeted intervention of drug resistance pathways are the key to improving the long-term prognosis of patients.

### 2.2. Current Status of Conventional Drug Therapy for CRC

Traditional drug treatments for CRC include three categories: chemotherapy, targeted therapy, and palliative therapy. Among chemotherapeutic agents, the classic antimetabolite 5-fluorouracil (5-FU) demonstrates efficacy but is limited by low cure rates, drug resistance, and cardiotoxicity. Irinotecan is metabolized to its active form SN-38 and benefits selected patients, yet it suffers from poor water solubility, low bioavailability, and frequent adverse reactions [16]. Oxaliplatin, a platinum-based alkylating agent, induces DNA damage and activates antitumor immunity but is associated with peripheral neuropathy, and its combination with 5-FU agents yields limited additional benefits. Capecitabine, a prodrug of 5-FU, offers convenient oral administration and low toxicity, serving as an alternative therapeutic option. Regorafenib, a multi-target tyrosine kinase inhibitor, is indicated mainly for metastatic patients with multiple treatment failures, requiring close monitoring for hypertension and bleeding risks. Clinically, combination chemotherapy enhances antitumor effects but is hindered by insufficient tumor specificity and the development of drug resistance.

Targeted agents fall primarily into three classes: dual anti-HER2 therapies, including trastuzumab and pertuzumab, achieve notable efficacy in genetically selected patients but frequently develop resistance due to gene mutations [17]. The anti-VEGF agent bevacizumab improves survival when combined with chemotherapy yet commonly induces hypertension. Epidermal growth factor receptor inhibitors cetuximab and panitumumab are restricted to specific wild-type patients, with risks of drug resistance and potential hypersensitivity reactions.

In palliative care, analgesia relies mainly on strong opioids such as methadone, morphine, and fentanyl. Methadone combined with morphine provides superior analgesia but requires professional supervision. Individualized use of antiemetics and nutritional support agents effectively improve quality of life in patients with advanced CRC.

## 3. Classification of PSHs

PSHs are hydrogel materials with a three-dimensional hydrophilic network structure formed by physical entanglement, covalent bonds or ionic bonds and other cross-linking methods, with polysaccharide macromolecules as the main framework. The core basis for their classification is the number and source of the constituent macromolecules, which can be divided into two major categories: single macromolecule PSHs and composite macromolecule PSHs. Under each category, they can be further classified based on the source, components, etc. The specific classification and characteristics are as follows:

### 3.1. Single-Polymer PSHs

These hydrogels use a single polysaccharide polymer as the sole substrate, forming a three-dimensional network through physical or chemical cross-linking. They are characterized by single component, clear structure, and controllable performance. Based on the source of polysaccharides, they can be divided into four categories: plant, marine, microbial, and animal. Each category has distinct advantages and application scenarios [18], with relevant literature examples listed in Table 1.

#### 3.1.1. Single-Polymer Plant PSHs

Using a single polysaccharide derived from terrestrial higher plants as the sole substrate, these hydrogels form a network via physical entanglement, hydrogen bonds, ionic interactions, or covalent bonds. Their core characteristics include good biocompatibility, biodegradability, wide availability, and environmental friendliness. Starch-based hydrogels are a typical system; after oxidation cross-linking, they can achieve sustained drug release and inhibit cancer cell proliferation, showing good therapeutic delivery potential. Cellulose-based hydrogels have adjustable mechanical properties and are mainly used in flexible sensors and drug-controlled release systems [19]. Gellan gum hydrogels are divided into high and low methoxy types, focusing on food encapsulation and active-substance controlled release [20]. Guar gum hydrogels efficiently adsorb Cu^2+^ and are applied in heavy metal wastewater treatment [21]. Other plant PSHs (e.g., inulin, arabinogalactan) exhibit potential in colon-targeted delivery and tissue engineering scaffolds.

#### 3.1.2. Single Polymer Marine PSHs

Using natural polysaccharides from marine algae as the sole substrate, these hydrogels form a network through non-covalent interactions or mild covalent cross-linking. Their core characteristics are excellent biocompatibility, complete biodegradability, rich functional groups in the molecular chain, as well as high hydrophilicity, high swelling, and biological activity. Sodium alginate hydrogels gel rapidly and are a classic choice for drug delivery and wound dressings. Carrageenan hydrogels have high mechanical strength, making them suitable for biosensing and tissue engineering scaffolds. Agar/agarose hydrogels have high transparency and stable structure and are mainly used for three-dimensional cell culture and biochemical separation [22]. Kelp polysaccharide hydrogels have antioxidant and immune regulatory activities, standing out in regenerative medicine [23]. Ulvan green algal polysaccharide hydrogels have excellent moisturizing and repair-promoting activities, showing potential in wound dressings and other fields [24]. These hydrogels are prepared under mild conditions and are non-toxic, facilitating clinical application.

#### 3.1.3. Single Polymer Microbial PSHs

Using homologous polysaccharides synthesized by microbial fermentation as the sole substrate, these hydrogels form a network through physical or chemical interactions. Their core characteristics are pure structure, clear composition, excellent biocompatibility, and high scalability for fermentation production. Compared with plant polysaccharides, they have greater advantages in greenness and controllability. Typical representatives include: xanthan gum hydrogels with high viscosity and shear thinning properties; dextran hydrogels with adjustable swelling and degradation behaviors [25]; chondroitin gum hydrogels with high strength and transparency [26]; pullulan hydrogels with high biological safety, meeting FDA-GRAS standards [27]; callose hydrogels with outstanding heat resistance; and microbial fermentation-type hyaluronic acid hydrogels that are injectable, self-repairable, and can closely simulate the extracellular matrix. These hydrogels have reliable application potential in wound dressings, drug carriers, and other fields.

#### 3.1.4. Single Polymer Animal PSHs

Using natural polysaccharides extracted from animal tissues and body fluids as the sole substrate, these hydrogels form a network through physical or chemical cross-linking. Their core characteristics are single component, pure structure, excellent biocompatibility, and biodegradability. The main components include chitosan, animal-derived hyaluronic acid, chondroitin sulfate, and glycogen. These hydrogels are mainly based on physical cross-linking, without residual toxic cross-linking agents, and have high biological safety. Their mechanical and degradation properties can also be regulated through mild chemical cross-linking. Due to their ability to best retain the inherent activity of polysaccharides, they are widely used in drug delivery, tissue engineering, and antibacterial dressings in the biomedical field [28].

**Table 1:** Classification, crosslinking methods and core applications of monomeric PSHs.

Single Macromolecular Polysaccharide	Polysaccharide Type	Physical Crosslinking	Chemical Crosslinking	Core Applications	References
Plant	Starch (derived from pea, potato)	Freeze-thaw physical crosslinking	Sodium periodate oxidation crosslinking; Schiff base chemical crosslinking (asparagine)	Therapeutic drug delivery, targeted regulation of oral squamous cell carcinoma	[29]
	Cellulose-based	ionic crosslinking; physical crosslinking	Chemical crosslinking (carboxymethylation, oxidative crosslinking)	Controlled drug delivery, oral administration, local drug release	[30]
	Pectin	High-methoxyl pectin: hydrogen bonding + hydrophobic interaction; low-methoxyl pectin: calcium ion “egg-box” ionic crosslinking; physical; crosslinking	Chemical; IPN	Food functionalization, active substance encapsulation, fat substitution, food packaging, 3D-printed food	[20]
	Guar gum	Ionic crosslinking	Chemical covalent crosslinking	Adsorption of heavy metal copper ions, water pollution treatment	[21]
	Inulin	Ionic crosslinking; physical blending crosslinking	Chemical crosslinking (methacrylation crosslinking)	Colon-targeted drug delivery, bioactive substance encapsulation, medical diagnostic imaging	[31]
	Arabinoxylan	-	Laccase-catalyzed ferulic acid oxidative covalent crosslinking	Colon-targeted insulin delivery, adjuvant therapy for diabetes mellitus	[32]
Marine	Alginate	Ionic crosslinking (calcium ions)	-	High water-retention materials, biomedical dressings	[33]
	Carrageenan	Physical crosslinking (potassium/calcium ions, double helix structure); ionic crosslinking	-	Food industry, biomedicine, drug delivery	[34]
	Agarose	Physical crosslinking (hydrogen bonding)	Chemical crosslinking	Tissue engineering, drug delivery, bioimaging	[22]
	Fucoidan	Ionic crosslinking; physical entanglement	Dynamic covalent bond crosslinking	Anti-inflammatory therapy, wound healing, injectable biomaterials	[23]
	Ulvan (Ulva polysaccharide)	Ionic crosslinking; physical crosslinking	chemical crosslinking; hybrid crosslinking	Biomaterial design, tissue engineering, wound healing	[35]
Microbial	Pullulan	Physical blending	TEMPO/periodate oxidation modification; chemical crosslinking with chitosan/gelatin	Wound healing; skin tissue engineering; drug delivery	[27]
	Gellan gum	Physical crosslinking (electrostatic interaction, hydrogen bonding, van der Waals forces)	-	Antibacterial materials; biomedical hydrogels; food applications	[36]
	Hyaluronic acid	Physical crosslinking (hydrogen bonding, electrostatic interaction, coordination interaction, host–guest recognition)	Chemical crosslinking (small molecule crosslinkers, macromolecular self-crosslinking, photocrosslinking, enzymatic crosslinking)	Drug delivery; cartilage repair; three-dimensional cell culture; skin dressings	[37]
	Xanthan gum	Polymer blending; physical reinforcement	Chemical crosslinking	Hydrogel toughening; adhesive biomaterials	[38]
	Salecan polysaccharide	Ionic crosslinking; physical blending	Chemical crosslinking	Biomedical engineering; drug delivery; tissue engineering	[39]
	Destrin polysaccharide	Physical crosslinking	-	Infected wound healing; antibacterial wound dressings	[40]
	Dextran	-	Chemical crosslinking; multiscale structural design	Tissue engineering scaffolds; microgels; nanofibers	[25]
	Levan (bacterial exopolysaccharide)	Physical crosslinking	Chemical modification	Biomedical materials; drug delivery; tissue engineering	[41]
Animal	Chitosan	Physical crosslinking (molecular entanglement, hydrogen bonding, ionic/hydrophobic interaction); crosslinking; coordination	Chemical; (covalent; bond,; photocrosslinking)	Drug delivery, tissue engineering, antibacterial/antifungal, injectable gels, water treatment (heavy metal/dye removal), molecular dynamics simulation of anticancer drug delivery	[42]
	Chondroitin sulfate	Ionic crosslinking; self-assembly	Chemical crosslinking; enzymatic crosslinking	Osteoarthritis treatment, tissue engineering scaffolds, drug delivery, functional foods, wound dressings	[43]
	Enzymatically synthesized glycogen	Self-assembly; crosslinking	Dodecyl; hydrophobic; modification; non-covalent	Protein refolding, artificial molecular chaperones	[44]

### 3.2. Composite Polymer PSHs

Composite polymer PSHs are formed by combining two or more polymer materials, at least one of which is a polysaccharide polymer. They form a composite three-dimensional network through methods such as co-crosslinking and IPN. Their core advantage is the ability to compensate for the performance shortcomings of single PSHs and achieve targeted regulation of structure and function. Based on the composition of composite components, they can be divided into five types, with relevant literature examples detailed in Table 2.

#### 3.2.1. Polysaccharide–Polysaccharide Composite Hydrogels

These hydrogels consist of two or more natural polysaccharides from different sources, without introducing non-polysaccharide components. Their core feature is the possession of biocompatibility, degradability, and structural tunability. Constructed through hydrogen bonds and ionic crosslinking, they effectively improve the brittleness and uncontrollable swelling of single gels and are widely applicable in food, biomedicine, and other fields. Based on the source of polysaccharides, they can be divided into seven composite types: inter-composites of plant polysaccharides, composites of plant and marine polysaccharides, composites of plant and microbial polysaccharides, inter-composites of marine polysaccharides, composites of marine and microbial polysaccharides, inter-composites of microbial polysaccharides, and animals combined with microorganisms. Each composite system achieves performance optimization through different mechanisms and is suitable for various application scenarios.

#### 3.2.2. Polysaccharide-Natural Non-Polysaccharide Polymer Composite Hydrogels

This type of hydrogel uses natural polysaccharides as the framework, assembled with protein-based natural polymers. Its core feature is the combination of polysaccharides’ hydrophilicity and degradability, and proteins’ high biocompatibility and weak immunogenicity. Through synergistic crosslinking, it compensates for the weak mechanical properties and rapid degradation of single-polysaccharide hydrogels, serving as a core carrier material in food and biomedicine [45]. Common combinations mainly fall into two categories: composites of polysaccharides and structural proteins, which can enhance cell adhesion and biomimetic structure, as well as improve mechanical strength and self-repair ability; and composites of polysaccharides and storage proteins, which are widely used in food gels, edible coatings, and active substance delivery. The driving force for the composite is mainly electrostatic interaction and hydrogen bonds, which is supplemented by ionic crosslinking to achieve structural stability. Its performance can be precisely controlled through component ratio and preparation process [46].

#### 3.2.3. Polysaccharide-Synthetic Polymer Composite Hydrogels

This type of hydrogel uses natural polysaccharides as the biocompatible framework combined with synthetic polymers. Its core advantage is integrating the biocompatibility of natural polysaccharides with the mechanical tunability and precise response of synthetic polymers, effectively compensating for the performance bottlenecks of single PSHs and becoming the mainstream design direction of biomedical soft materials. Common synthetic polymers include PVA, PAM, PEG, etc. They form a stable network through physical entanglement and covalent crosslinking. After composite modification, the hydrogel’s mechanical strength, stability, and anti-fouling ability can be significantly improved, and functions such as swelling regulation, controlled drug release, and degradation rate regulation can also be achieved. It is widely used in drug delivery, tissue engineering, and other fields [47].

#### 3.2.4. Polysaccharide-Inorganic Nanomaterial Hybrid Hydrogels

This type of hydrogel uses natural polysaccharides as the organic framework and introduces inorganic nanophases. Its core feature is the synergistic improvement of mechanical properties, biological activity, and stimulus-responsive functions, breaking through the limitations of weak mechanical properties and single-functionality of single polysaccharide systems, becoming an important research direction in biomedical hydrogels. Different inorganic nanophases endow the system with differentiated functions: clay/vermiculite increases water absorption rate and stability; nano-hydroxyapatite constructs biomimetic bone matrix scaffolds [48]; graphene nanoparticles provide photothermal properties and conductivity [49]; Fe_3_O_4_ magnetic nanoparticles realize magnetic targeted drug delivery and magnetic heat synergistic treatment [50]; and silver nanoparticles have both controlled release and broad-spectrum antibacterial functions. Various composite systems can adapt to scenarios such as hygiene materials, bone tissue engineering, wound repair, and tumor treatment [51].

**Table 2:** Classification and characteristics of composite PSHs.

Composite Type	Polysaccharide Category	Specific System	Physical Crosslinking	Chemical Crosslinking	Core Applications	References
Polysaccharide-Polysaccharide Composite	Plant–Plant	Pectin–starch	Ca^2+^ crosslinking (egg-box structure), intermolecular hydrogen bonding, electrostatic interactions	-	Functional foods, prebiotic carriers, bioactive delivery systems, food packaging/additives	[52]
	Plant–Marine	Locust bean gum–κ-carrageenan	Hydrogen bonding, electrostatic interactions, molecular entanglement, phase separation and protein network construction	-	Dairy gels, food texture modification, protein–polysaccharide composite systems	[53]
	Plant–Microbial	Salep glucomannan–xanthan gum	Hydrogen bonding, helical conformation synergy, physical entanglement	-	pH/thermo-sensitive hydrogels, food gels, biomedicine	[54]
	Marine–Marine	Fucoidan–κ-carrageenan	Hydrogen bonding, molecular chain entanglement, κ-carrageenan helical aggregation	-	Foods, bioactive carriers, gel microbeads, fibers, films	[55]
	Marine–Microbial	Oxidized dextran–sodium alginate	Hydrogen bonding	Schiff base linkages, dynamic covalent bonds	Self-healing hydrogel dressings, hemostasis, burn wound healing	[56]
	Microbial–Microbial	Gellan gum–xanthan gum	Hydrogen bonding synergy, physical blending crosslinking	-	Plant-based enteric films, enteric hard capsules, drug/functional food coatings	[57]
	Animal–Microbial	Chondroitin sulfate–hyaluronic acid	Molecular chain entanglement, biocompatible crosslinking	Non-covalent interactions	Cartilage repair, anti-inflammatory hydrogels, articular tissue engineering	[58]
Polysaccharide-Natural Non-Polysaccharide Polymer Composite	Polysaccharide–Structural Protein	Alginate–collagen	Synergy of divalent cation ionic crosslinking and physical crosslinking	-	Tissue engineering, tissue regeneration, wound dressings and cell therapy	[59]
	Polysaccharide–Storage Protein	Gellan gum–soy protein isolate	-	Cation-induced and hydrogen-bonded non-covalent self-assembly	Sustained and controlled release of active substances, hypoglycemic functional carriers	[60]
Polysaccharide-Synthetic Polymer Composite	Polysaccharide–PVA	Tamarind seed xyloglucan–PVA	Freeze-drying physical pore formation	Citric acid chemical crosslinking	Drug delivery, wound dressings, hygiene products	[61]
	Polysaccharide–PAM	Platycodon grandiflorus polysaccharide–PAM	Ca^2+^-mediated self-assembly	Schiff base reaction	Wound dressings, wound healing	[62]
	Polysaccharide–PEG	Hyaluronic acid –PEG–p(HPMAm-lac)	Thermogelation	Michael addition dual crosslinking	Cell carriers, controlled drug release, tissue engineering	[63]
	Polysaccharide–Acrylic Acid	Xylan-rich hemicellulose–acrylic acid	-	N’-Methylenebisacrylamide chemical cross-linking	Adsorption separation, drug delivery systems	[64]
	Polysaccharide–HPMC	β-Cyclodextrin–HPMC	-	Citric acid chemical crosslinking	Hydrophobic drug delivery	[65]
Polysaccharide-Inorganic Nanoparticle Hybrid	Polysaccharide + Montmorillonite	Starch–montmorillonite	-	UV-initiated free radical polymerization, chemical crosslinking crosslinker)	pH-responsive controlled release delivery of neuroprotective drugs (citicoline)	[66]
	Polysaccharide + Bentonite/Silica Particles	Carboxymethyl guar gum–bentonite	Physical filling reinforcement	Chemical crosslinking	Disposable hygiene products (superabsorbent gels)	[67]
	Polysaccharide + Nano-Hydroxyapatite	Natural polysaccharide–hydroxyapatite	In situ composite, physical coating	-	Efficient cancer therapy (nuclear-targeted nanocarriers)	[48]
	Polysaccharide + Graphene Nanoparticles	Gelatin, chondroitin sulfate–graphene nanoplatelets (xGnP)	Physical blending, electrostatic interactions	-	Drug delivery, tissue engineering (cartilage repair)	[49]
	Polysaccharide + Fe_3_O_4_ Magnetic Nanoparticles	Alginate, chitosan, cellulose, starch, agarose, hyaluronic acid–magnetic nanoparticles (Fe_3_O_4_, γ-Fe_2_O_3_, CoFe_2_O_4_)	Physical blending, ionic crosslinking	In situ precipitation, grafting chemical cross-linking	Targeted drug delivery, tissue regeneration, magnetothermal therapy	[50]
	Polysaccharide + MoS_2_ Nanosheets	Polysaccharide–molybdenum disulfide nanosheets	Dynamic crosslinking, self-healing crosslinking	-	Synergistic photothermal–photodynamic therapy for breast cancer	[68]
	Polysaccharide + Silver Nanoparticles (SNPs)	Guar gum–silver nanoparticles	-	Graft polymerization, chemical crosslinking	Biomedicine (antibacterial, drug delivery)	[51]
	Polysaccharide + Polyhedral Oligomeric Silsesquioxane (POSS)	Chitosan–POSS	Physical interactions	Chemical crosslinking	Bone tissue engineering	[69]
	Polysaccharide + CuS Nanoparticles	Polysaccharide-CuS, DOX@ZIF–8 nanoparticles	-	Dynamic covalent crosslinking, Schiff base reaction	Synergistic photothermal–photodynamic–chemotherapy for cancer	[70]
Composite of Three or More Polysaccharides	Polysaccharide + Polysaccharide + Polysaccharide	Cationic starch–κ-carrageenan–sodium alginate	Physical crosslinking: electrostatic interactions between cationic groups of starch and anionic groups of carrageenan/sodium alginate, molecular chain entanglement, hydrogen bonding of hydroxyl groups, K^+^, Ca^2+^ promoted gelation of carrageenan and sodium alginate	-	Biosensors, controlled drug release, food processing, biomedical engineering, adsorption removal of cationic dyes (methylene blue)	[71]

#### 3.2.5. Three or More Polysaccharides Composite Hydrogels

Multi-component polysaccharide composite hydrogels rely on the synergistic effect of multiple components to make up for deficiencies in mechanical strength, stability, and biological functions of single and binary systems, becoming a research hotspot in CRC, drug delivery, and tissue engineering. This type of material is usually made from common polysaccharides such as chitosan, sodium alginate, hyaluronic acid, and cellulose. Through electrostatic interaction, hydrogen bonds, ionic crosslinking, and dynamic covalent bonds, it forms an interpenetrating network to achieve significant improvement in comprehensive performance. Typical systems, after modification, can possess temperature/pH dual-response, excellent antibacterial properties, good biocompatibility, or high strength, making them suitable for various application requirements such as adsorption, 3D bioprinting, and soft tissue regeneration. These hydrogels can also be combined with AI to optimize preparation processes and enhance targeting and can load 5-FU to improve therapeutic effects in CRC by responding to TME.

## 4. Types of Polysaccharide Hydrogel Responses

### 4.1. The TME of CRC and the Classification of Responsive PSHs

The TME of CRC is a complex and dynamic system composed of cellular components, non-cellular components, and signaling molecules. It exhibits high heterogeneity and directly regulates tumor proliferation, invasion, metastasis, and therapeutic resistance [72]. Its typical pathological characteristics include an acidic microenvironment, high accumulation of reactive oxygen species (ROS), immunosuppression, local hypoxia, and a dense ECM barrier. Tumor cell metabolic reprogramming and oxidative stress respectively lead to issues such as lactic acid accumulation and excessive ROS production. Matrix remodeling hinders drug penetration [73]. Abnormal activation of signaling pathways, including transforming growth factor-β (TGF-β), phosphatidylinositol 3-kinase (PI3K)-Akt-mammalian target of rapamycin (mTOR), and programmed cell death protein 1 (PD-1)/programmed death-ligand 1 (PD-L1), further promotes tumor progression. Based on these characteristics, natural polysaccharide hydrogels can be classified according to their response mechanisms (as shown in Table 3 and Table 4). By targeting and regulating the TME, they can achieve efficient treatment, providing diversified new strategies for precise treatment of CRC.

### 4.2. Thermosensitive Hydrogel

The therapeutic application of thermosensitive hydrogels is focused on the subtype of CRC with peritoneal metastasis, which occurs in approximately 1/5 of CRC cases [74]. In traditional systemic chemotherapy for this subtype, the local drug concentration in the peritoneum is only 8% to 35% of the blood concentration, leading to limited therapeutic efficacy and significant systemic toxicity [75]. To address this issue, thermosensitive hydrogels, featuring core advantages of “local retention, precise controlled release, minimally invasive safety, and synergistic enhancement,” provide a novel local chemotherapy strategy for this subtype [76].

These hydrogels, based on materials such as methylcellulose and poly(lactic-co-glycolic acid)/polyethylene glycol (PLGA/PEG) block copolymers, exist in a solid state at room temperature, facilitating minimally invasive injection into the abdominal cavity. Upon entering the body, they rapidly undergo in situ gelation at the physiological temperature (37 °C), forming a stable drug reservoir in the abdominal cavity. They can also load chemotherapeutic drugs (e.g., 5-Fu, oxaliplatin) through physical embedding and other methods, achieving controlled sustained release for several days to weeks. This not only significantly increases the local drug concentration but also reduces systemic adverse effects. Additionally, the minimally invasive administration of these hydrogels ensures uniform distribution; they possess excellent biocompatibility and degradability and can also exert synergistic sensitization effects by regulating the TME, thereby helping to overcome tumor drug resistance.

Animal experiments have confirmed that after 21 days of treatment, the number of tumor nodules decreased by 75.3% ± 4.2%, the tumor volume was reduced by 69.5% ± 3.5%, the median survival time was prolonged by 81.3%, and the body weight of the subjects remained stable, with a significant reduction in myelosuppression [77]. Currently, this therapeutic strategy is still in the preclinical stage of clinical translation and faces challenges such as poor matching of degradation rates and insufficient deep penetration of drugs in large lesions. These issues need to be further verified for safety and efficacy through Phase I/II clinical trials [78].

### 4.3. pH-Responsive Hydrogels

pH-responsive hydrogels focus on the needs of precise local treatment and are applicable to the treatment of CRC as well as other diseases. With the core advantages of “microenvironment responsiveness, targeted delivery, multifunctional synergy, and biosafety”, they have become a potential strategy for the local treatment of refractory diseases [79]. These hydrogels are constructed based on pH-sensitive groups and acid-sensitive crosslinking structures, using carboxymethyl chitosan and alginate as matrix materials. They can sense pathological acidic microenvironments such as the TME (pH 6.0–6.5) and the colon (pH 6.5), triggering gel swelling, degradation, and drug release to achieve on-demand drug delivery.

pH-responsive hydrogels can load drugs such as methotrexate (MTX) and aspirin (Asp) through physical encapsulation, with adjustable release rates, excellent biocompatibility, and degradability, enabling multiple therapeutic effects suitable for oral and local administration. To address pain relief in CRC chemotherapy, an oral alginate–carboxymethyl cellulose (CMC)-CaCO_3_/MTX/Asp composite has been developed for colon-targeted release, achieving a synergistic effect of chemotherapy and pain relief. In vitro studies have shown that this composite exhibits a better inhibitory effect on SW480 colon cancer cells than free drugs while causing less damage to normal cells. Currently, most of these hydrogels remain in the preclinical stage, facing challenges such as a broad pH response range that may lead to drug leakage, difficulty in large-scale production, and lack of clinical validation, urgently requiring promotion toward clinical translation [80].

### 4.4. Photosensitive Hydrogel

Photosensitive hydrogels serve as an innovative supplementary strategy for the precise local treatment of CRC, focusing on the therapeutic needs of primary lesions, postoperative residual lesions, and local metastatic lesions. With the core advantages of “light-controlled targeting, multi-effect synergy, minimally invasive adaptability, and low toxicity with high efficiency”, they effectively address the drawbacks of traditional treatments. Their therapeutic characteristics are highly consistent with clinical needs: near-infrared (NIR) light at 808 nm and 660 nm is used as the excitation source, combined with photoreactive units such as azobenzene and pyran, and drug release is remotely regulated through photoreactions, achieving precise spatiotemporal control and reducing damage to normal intestinal tissues [81]; integrating PTT, chemotherapy, and immunotherapy, photothermal components such as MoS_2_ and 5-FU can also activate immune pathways to convert “cold tumors” into “hot tumors”; alginate and chitosan are used as matrix materials, and the aldehyde-amino reaction is employed to enhance the colonization ability in intestinal tumors, with excellent biocompatibility and degradability [82]; drugs such as 5-FU and 5-aminolevulinic acid (ALA) can be loaded through physical embedding or chemical bonding, and the proportion of photoreactive units can be adjusted to optimize therapeutic effects.

Therapeutic administration options include local injection (e.g., AlgNB/MoS_2_/5-FU hydrogel) or intestinal administration. In vitro experiments have demonstrated that photosensitive hydrogels can inhibit the proliferation of SW480 and CT26 colon cancer cells [83]. In the Balb/c mouse model, chitosan-based hydrogel (PS2-PEG-CS) loaded with m-tetrahydroxyphenylchlorin (m-THPC) derivatives (PS2) was found to achieve complete tumor regression after multiple light irradiations, and 75% of the cured mice were able to reject the secondary challenge of CT26 cells [84]. Currently, this therapeutic strategy is in the preclinical stage, facing challenges such as limited light penetration due to intestinal wall thickness, the need for a material degradation rate to match the 2–3 week chemotherapy cycle, and insufficient drug penetration in large-volume tumors. The optimal parameters and applicable population need to be clarified through Phase I/II clinical trials.

### 4.5. Enzyme-Responsive Hydrogels

Enzyme-responsive hydrogels represent a potential strategy for local chemotherapy and synergistic treatment of CRC, with core advantages of “enzyme-controlled targeting, precise sustained release, minimally invasive safety, and metastatic adaptability” [85]. Their carriers typically include natural polysaccharides such as inulin, carboxymethyl chitosan, and rhamnogalacturonan I, as well as biocompatible synthetic polymers such as polymethylacrylate. These hydrogels are triggered to release drugs by specific enzymes, including those highly expressed in the CRC TME, intestinal lysozyme, and metabolic enzymes of colonic beneficial bacteria. In normal tissues, they maintain a stable structure and only degrade at the tumor site, thereby significantly reducing systemic toxicity. By optimizing the concentration of matrix metalloproteinase (MMPs)-sensitive peptides, the ratio of polysaccharides, and other parameters, long-term sustained release for several days to several weeks can be achieved. Some enzyme-responsive hydrogels exhibit a dual-phase release characteristic of “rapid surface release—slow deep release”, which avoids the “peak–valley effect” associated with traditional chemotherapy.

Treatment regimens can be flexibly designed according to lesion characteristics: oral administration is suitable for primary colon lesions, intratumoral injection for primary lesions and liver metastases, and intraperitoneal injection for peritoneal metastases; the dosage can be adjusted based on tumor burden. For example, the inulin-based hydrogel loaded with oxaliplatin, after oral administration, is degraded by the metabolic enzymes of colonic beneficial bacteria and produces short-chain fatty acids (SCFAs), which significantly inhibits tumor growth; the carboxymethyl chitosan hydrogel loaded with imatinib, triggered by intestinal lysozyme for drug release, has a tumor inhibition rate six times higher than that of traditional preparations. Currently, relevant research has achieved significant results in in vitro experiments and animal models but still faces challenges: the specificity of enzyme response needs to be further improved to reduce drug leakage, drugs exhibit insufficient deep penetration in solid tumor lesions larger than 5 mm, the matching between material degradation rate and clinical chemotherapy cycle needs optimization, and large-scale clinical trials are lacking for verification. In the future, these bottlenecks can be gradually overcome through material modification, administration regimen optimization, and clinical translation research [86].

### 4.6. ROS-Responsive Hydrogels

ROS-responsive hydrogels, with ROS-sensitive groups such as thioacetals and borate esters as core response units, can achieve controlled drug release at tumor sites and integrate multiple mechanisms, including chemotherapy, immune regulation, and ferroptosis induction. This not only effectively inhibits tumor growth but also significantly reduces the systemic toxicity of traditional treatment regimens, providing a safe and efficient new treatment strategy for difficult-to-treat CRC such as immune-suppressed and chemotherapy-resistant types [87,88].

Their advantages are prominent: they can precisely identify the high-ROS TME, degrade only at the lesion site to achieve targeted drug release, and reduce damage to normal tissues. They possess intelligent self-catalytic release characteristics, promoting ROS generation after drug release to form a positive feedback loop, enhancing utilization and supporting dual-drug co-delivery; they can be administered as intratumoral injections to form long-lasting reservoirs or formulated as oral agents targeting the colon, offering both sustained release and gastrointestinal stability, and are made from biodegradable materials such as hyaluronic acid and polyglutamic acid, with excellent biocompatibility and safety.

In terms of therapeutic efficacy, ROS-responsive hydrogels show remarkable performance: the dual-drug delivery system achieved tumor growth inhibition rates of 56.6% and 68.9% in CT26 and MC38 models, respectively, and the combination with immune checkpoint inhibitors further enhanced the anti-tumor effect, effectively prolonging the survival of tumor-bearing mice; they can reverse the immunosuppressive microenvironment, increase the infiltration of CD8^+^T cells and neutrophils, promote the maturation of dendritic cells and the polarization of M1-type macrophages, and increase the levels of IFN-γ and perforin; they induce ferroptosis in colorectal cancer cells through the NAD^+^/Stat3/Gpx4 signaling axis, effectively sensitizing MSS-type colorectal cancer that is resistant to chemotherapy and insensitive to immunotherapy; compared with free drugs, they significantly reduce liver and kidney toxicity and gastrointestinal damage and support both injection and oral administration, allowing for targeted drug delivery within the tumor or oral administration to regulate the intestinal flora, with good potential for clinical translation [89].

### 4.7. Electro-Responsive Hydrogels

Electro-responsive hydrogels are a type of intelligent material that can undergo swelling, contraction, or structural deformation under an electric field. They provide innovative strategies for the local treatment and recurrence prevention of CRC. Their core characteristics include: precise controlled release, which can achieve “switchable” pulsed drug administration by adjusting voltage, electric field intensity, and duration, making them suitable for treatment at different stages. For example, the GelMA/κ-carrageenan/rGO composite hydrogel can achieve controllable drug release under a low voltage of 1 V [90]; minimally invasive compatibility, as most are injectable and can be administered through minimally invasive methods such as endoscopy and puncture, fitting well with cavities and wounds, with excellent biocompatibility and degradability; synergistic enhancement, as they can carry platinum-based components, achieving triple synergy of chemotherapy, electrocatalytic tumor killing, and activation of anti-tumor immunity under an electric field; strong targeting, as they form in situ and localize at the lesion site, reducing systemic exposure and systemic toxicity.

In basic and animal experiments, conductive composite hydrogels such as GelMA/rGO and sodium alginate/polypyrrole can effectively load drugs such as 5-FU and MTX and achieve stable drug release and inhibition of cancer cell proliferation under low-voltage electric fields [91]. Among them, the sodium alginate/polypyrrole hydrogel in mouse models, combined with electrocatalysis and chemotherapy, achieved a tumor inhibition rate of 83% without obvious liver, kidney, or intestinal damage. Currently, the therapeutic strategy has developed into a combined electrocatalysis–chemotherapy–immunotherapy approach, enhancing the depth of local treatment. These hydrogels are suitable for various clinical scenarios and possess multiple auxiliary functions. Although they have not yet entered large-scale clinical application, they have become one of the most translationally valuable directions for intelligent local drug delivery systems in CRC, providing feasible solutions for clinical challenges such as postoperative recurrence and chemotherapy resistance [92].

### 4.8. Magnetic-Responsive Hydrogels

The magnetic-responsive hydrogel is based on biocompatible polymers and composites manganese–zinc ferrite and other superparamagnetic nanoparticles. Under an external alternating magnetic field, it generates a magnetic heat effect, enabling the controlled release of drugs and local thermal ablation of tumors simultaneously. Compared to traditional treatment methods, it can achieve in situ tumor fixation, precise thermal ablation, and local drug sustained release, significantly reducing systemic toxic side effects, and providing a minimally invasive and highly efficient new treatment plan for advanced, unresectable, or postoperative prone-to-recurrence colorectal cancer. This hydrogel has both temperature-sensitive and magnetic-responsive characteristics. At room temperature, it is in a solid state and is convenient for injection or rectal administration. It can rapidly gel in the body to achieve precise retention of the lesion [93].

Under the magnetic field, the magnetic nanoparticles generate heat through hysteresis loss and Néel relaxation, causing the hydrogel to contract and pulse drug release, enabling a multi-mode collaborative treatment system. In addition, the alternating magnetic field has the advantages of deep tissue penetration and non-invasive regulation, avoiding the risk of intestinal perforation, and its matrix is degradable, non-toxic, and can adhere to the inner wall of the intestinal cavity, prolonging the treatment duration.

Currently, these hydrogels have achieved good preclinical results: In locally advanced rectal cancer, injectable magnetic thermosensitive hydrogel can be administered via rectal or endoscopic injection, and under the magnetic field, it is heated to above 50 °C to achieve thermal ablation. A single treatment reduces the tumor volume by more than 60%, helping to achieve preoperative tumor downstaging and preservation of the anus; in the in situ colon cancer model, the NO-releasing magnetic-responsive hydrogel can enhance mild magnetic thermal efficacy, achieve complete tumor clearance without significant recurrence; the new structural hydrogel can achieve colon-targeted drug delivery, reducing the early release of drugs in the stomach. In summary, the magnetic-responsive hydrogel provides a new technical path for precise, minimally invasive, and individualized treatment of CRC [94,95].

### 4.9. Multi-Sensory Responsive Hydrogels

Multi-responsive hydrogels are a novel intelligent delivery system for precise treatment of CRC, which can simultaneously respond to endogenous stimuli (pH, GSH, enzymes, ROS) and exogenous stimuli (temperature, near-infrared light, magnetic field) to achieve controlled and spatially controllable drug release [87]. This system is based on biocompatible polymers such as hyaluronic acid, alginate, and chitosan and is equipped with drugs such as oxaliplatin, 5-fluorouracil, and curcumin, possessing injectable, sprayable tissue adhesion and long-term sustained-release advantages. It can be used for local drug administration in postoperative residual lesions and can also achieve oral colon-targeted drug delivery, effectively improving problems such as poor targeting, high toxic side effects, and easy recurrence and metastasis of traditional chemotherapy [96].

Its core features are reflected in four aspects: The first is precise response. Through multiple signals such as pH + reduction and pH + photothermal, it can precisely identify the CRC microenvironment and rapidly release drugs at the lesion site. The second is synergistic enhancement. It can simultaneously carry chemotherapy drugs, photothermal agents, and immune stimulators to achieve combined chemotherapy–photothermal–immune therapy, inducing immunogenic cell death. The third is long-term local effect. It has strong tissue adhesion and can continuously release drugs for more than 20 days, maintaining an effective local drug concentration. The fourth is low toxicity and safety. The carrier can be degraded, and the drugs are released only at the lesion site, reducing systemic side effects [97].

Currently, this type of hydrogel has covered four clinical scenarios such as postoperative adjuvant therapy and prevention of abdominal metastasis. Sprayable pH/photothermal dual-responsive hydrogel can completely eliminate residual cancer after surgery, and animal experiments showed no recurrence within 80 days and a 100% survival rate. Redox/near-infrared light dual-responsive injectable hydrogel has a better tumor suppression rate for HT-29 and CT26 colon cancer cells than free drugs. pH/intestinal flora dual-responsive polysaccharide-based hydrogel can be orally targeted to the colon and enhance the therapeutic effect of MSS-CRC [98]. The thermosensitive/pH dual-responsive in situ gel system has entered the clinical transformation exploration stage, providing a highly potential new strategy for local precise treatment of colorectal cancer.

**Table 3:** Classification and Properties of responsive PSHs for CRC therapy.

Responsive Type of Polysaccharide Hydrogel	Polysaccharide Composition	Specific Targeted CRC Type	Application Advantages	References
Thermosensitive hydrogel	Chitosan, hyaluronic acid, sodium alginate, etc.	CRC	Local controlled release, reduced systemic administration, improved drug bioavailability, biodegradable, suitable for postoperative anti-recurrence and immunotherapy	[99]
	Methylcellulose	With peritoneal metastasis	Injectable in situ gelation; biocompatible and biodegradable; local long-term drug release; inhibits tumor proliferation and promotes apoptosis	[76]
	Poloxamer P407/P188, sodium alginate	CT26 model	Injectable at room temperature, gels at body temperature; shear-thinning; local sustained release of 5-FU; reduces systemic toxicity and inhibits recurrence	[77]
pH-responsive hydrogel	Chitosan, hyaluronic acid, sodium alginate, dextran, cellulose derivatives	Solid tumor	Targeted controlled release, excellent biocompatibility, high drug loading, injectable, self-healing, adaptive to acidic TME	[81]
	Alginic acid, hyaluronic acid, carboxymethyl cellulose, chitosan	Gastrointestinal tumors	Tunable structure, sensitive pH response, applicable for targeted drug delivery, sensing, separation, tissue engineering	[100]
	Alginate, sodium carboxymethyl cellulose	-	Dual-drug synergistic delivery, pH-controlled release, good biodegradability, reduced side effects, enhanced anti-tumor efficacy	[80]
	Okra polysaccharide, sodium alginate	-	Antibacterial and antioxidant, pH-sensitive drug release, excellent biocompatibility, nanocomposite structure improves drug loading and delivery efficiency	[101]
	Agarose, succinoglycan	-	Flexible and deformable, precise pH-controlled release, tunable mechanical properties, adaptable to complex in vivo environments	[102]
	Polysaccharide-based composite hydrogel	CRC (glutathione-overexpressing type)	Injectable, targeted to TME, dual-responsive synergistic therapy, precise drug release	[103]
	Carboxymethyl dextran	(Solid tumor)	Superabsorbent, pH-intelligent controlled release of anticancer drugs, biodegradable, high drug loading capacity	[104]
	Alginate, carboxymethyl cellulose, chitosan	Postoperative gastrointestinal perforation	Sprayable and easy to use; strong tissue adhesion for rapid perforation repair; good biodegradability to promote tissue healing; stable in gastrointestinal physiological environment	[105]
Photosensitive hydrogel	Sodium alginate	SW480 cell-derived, CT26 cell-derived	Injectable in situ gelation with strong tissue adhesion to prevent detachment; MoS_2_ provides high-efficiency photothermal effect, synergistic with 5-FU chemotherapy; inhibits tumor DNA repair and activates anti-tumor immunity; good biocompatibility and low in vivo toxicity	[85]
Enzyme-responsive hydrogel	Pectin	Ulcerative colitis (UC, colorectal inflammatory lesion)	Colon-targeted delivery of budesonide, reduced systemic side effects, excellent sustained drug release	[106]
	Carboxymethyl chitosan	CRC (solid tumor)	Oral delivery of hydrophobic imatinib, opens epithelial tight junctions to improve intestinal permeability, 6-fold enhancement of anti-tumor efficacy	[86]
	Inulin	(Orthotopic/subcutaneous solid tumor)	Modulates gut/tumor microbiome, activates anti-tumor immunity, enhances chemotherapy efficacy, good gastrointestinal stability	[9]
ROS-responsive hydrogel	Hyaluronic acid	Intestinal tissues at risk for colitis-associated	Protects probiotics from harsh gastrointestinal environments; targeted and precise bacterial release at inflamed colon sites; scavenges ROS to alleviate inflammation and protect intestinal mucosa; excellent biocompatibility with no obvious side effects	[107]
	Inulin	Orthotopic CT26, subcutaneous CT26	Oral administration with high patient compliance; modulates gut and intratumoral microbiome; generates short-chain fatty acids to activate anti-tumor immunity; prolongs colonic retention and enhances chemotherapy via targeted drug release	[9]
Electro-responsive hydrogel	Chitosan, alginate, dextran, carrageenan, etc.	-	Enables local controlled release at tumor sites, reduces systemic chemotherapy toxicity, suitable for postoperative residual tumors/unresectable tumors	[108]
Magneto-responsive hydrogel	Carboxylated cellulose nanofibers	Advanced, low rectal cancer (sphincter-preserving)	Rapid swelling, high mechanical strength, local precise drug delivery, synergistic magnetothermal/chemotherapy/chemodynamic therapy, preserves anal function	[95]
	Galactomannan, sodium alginate	(HT-29 cell model)	Green synthesis, pH-targeted controlled release, antioxidant/antibacterial activities, good biocompatibility, high drug encapsulation efficiency	[101]
	Hyaluronic acid, cellulose, sodium alginate, chitosan, pectin, galactomannan, etc.	(Including colon cancer, rectal cancer)	High targeting, controlled drug delivery, reduced systemic side effects, compatible with multiple administration routes, synergistic therapy	[109]

**Table 4:** Classification of multi-stimuli-responsive PSHs for CRC therapy.

Responsive Type of Polysaccharide Hydrogel		Polysaccharide Composition	Specific Targeted Type	Application Advantages	References
Multi-stimuli-responsive hydrogel	pH/photothermal dual-responsive	Hyaluronic acid, polydopamine	(Postoperative residual, peritoneal metastasis)	Sprayable tissue adhesive; stepwise pH-responsive drug release; synergistic chemotherapy + photothermal + immunotherapy; long-term in vivo drug release for 20 days; low toxicity and high efficiency, 100% recurrence-free survival in mice	[97]
	Thermo/pH, redox/pH, multi-stimuli-responsive	NIPAM, chitosan, carboxymethyl chitosan, dextran, etc.	(HT-29, HCT 116, etc.)	Adaptable to TME; precise controlled release; reduced systemic toxicity; enhanced drug targeting	[110]
	Glutathione/ROS dual-responsive	Chitosan	Peritoneal metastasis (CT26 cells)	Synergistic hyperthermic perfusion chemotherapy to improve drug uptake; precise TME-responsive drug release with high safety; inhibits HSP90 to reduce drug resistance; suppresses tumor angiogenesis with significant anti-metastasis effect	[111]
	ROS/gastrointestinal pH/enzyme/microbial-dual-responsive	Oxidized hyaluronic acid, gelatin, polydopamine	Colitis-associated (inflammatory CRC)	Oral delivery to inflamed colon sites, precise targeting of M1-type macrophages; multi-stage stepwise release, resistant to gastric acid and intestinal site-specific drug release; NO-mediated selective antibacterial activity, regulates gut microbiota and increases probiotic abundance; scavenges ROS, repairs intestinal barrier, inhibits inflammatory pathways and reduces cancer risk; good biocompatibility without obvious systemic toxicity	[112]
	TME-responsive (pH/enzyme-dual-responsive)	Dextran, alginate	Orthotopic	In situ gel formation for long-term sustained release of chemotherapeutic drugs; modulates tumor immune metabolism and activates immune response; reduces systemic chemotherapy toxicity; synergistic therapy inhibits tumor growth and recurrence	[113]

### 4.10. Combined Treatment with PSHs and Medications

The combined application of PSHs with chemotherapy drugs and natural active drugs has evolved from a simple sustained drug release carrier in the early stage to a comprehensive CRC precision treatment system that integrates functions such as intestinal targeting delivery and microenvironment-responsive drug release, achieving a leap from “passive drug loading” to “active treatment”. In the early stage, natural polysaccharides such as chitosan and alginate were used as the base materials to construct basic hydrogels, with the core function being drug sustained-release and protection of gastrointestinal stability; subsequently, new hydrogels with temperature-sensitive, pH/ion-responsive, etc., properties emerged, upgrading the carrier functions and expanding the treatment strategies to include chemotherapy combined with immune regulation, providing a new direction for colorectal cancer treatment [96].

At present, a diversified combination system has been established: lycium barbarum polysaccharide hydrogel loaded with oxaliplatin can achieve colon-targeted drug release, enhance tumor drug accumulation and regulate macrophage polarization; carboxymethyl chitosan nano-hydrogel co-loaded with oxaliplatin and resveratrol can synergistically kill tumors and reverse immunosuppression [114]; dextran/dahlia sugar hydrogel and purslane polysaccharide—alginate hydrogel beads and β-cyclodextrin hydrogel, respectively—achieve precise delivery of 5-FU through bacterial response, pH response, and mucosal adhesion; carboxymethyl cellulose composite hydrogel loaded with drugs such as methotrexate improves colon-targetedness [115]; inulin–pectin hydrogel regulates the intestinal flora during drug release [116]; chitosan-based nano-composite hydrogel can efficiently deliver prodrugs and improve the TME.

The core advantage of this strategy lies in the following: polysaccharide materials are safe and biodegradable, and multiple responses achieve precise drug release and reduction of chemotherapy side effects; it can be flexibly combined with drugs, has multiple functions, and is suitable for various clinical scenarios [117]. However, there are still issues such as insufficient mechanical stability, the impact of individual differences in the microbiota on efficacy, the need for optimization of multi-drug ratios, and difficulties in large-scale production. In the future, breakthroughs through material modification and other directions are needed to promote it to become a standardized treatment plan.

### 4.11. Clinical Application of PSHs

In the clinical application of PSHs for CRC, the core requirements are “minimally invasive adaptation, local efficacy, safety and controllability”. Currently, it is in the critical stage of transitioning from preclinical to clinical trials, with some systems having entered early clinical trial exploration. In early preclinical research, polysaccharides such as alginate and chitosan were mostly used as passive drug delivery scaffolds. Their drug release mainly relied on enzymatic hydrolysis by colonic bacteria or simple diffusion, lacking active targeting ability and responsiveness to the TME and failing to be optimized for the pathological characteristics of CRC [118].

With the continuous advancement of clinical needs, polysaccharides such as gelatin and hyaluronic acid have been widely used to construct injectable and in situ gelation systems. Relevant cell and animal experiments have achieved phased results: for example, gelatin composite hydrogels can synergistically load curcumin and 5-FU, significantly inhibiting the proliferation of CRC cells and providing a solid experimental basis for local chemotherapy of CRC; representative achievements include alginate microspheres encapsulating cabazitaxel nanoparticles and methylcellulose thermosensitive hydrogels carrying oxaliplatin [119]. These preparations can achieve high local drug concentration accumulation in tumors and effectively reduce the systemic toxic and side effects of drugs, laying the foundation for subsequent clinical transformation [76].

Based on the accumulation of previous experimental research, current clinical trials related to PSHs have covered multiple diseases. Youxi Zhou et al. reviewed the registration numbers and related applications of core clinical trials as follows: a clinical trial for the prevention and treatment of grade 2 or above radiation-induced skin damage in breast cancer patients (registration number: NCT04481802); a clinical trial for the treatment of diabetic wounds (registration number: NCT06492811); a clinical trial for accelerating the healing of chronic diabetic foot ulcers (registration number: NCT06584617), etc. [120]. Additionally, it was learned from ClinicalTrials.gov on 10 April 2026 that a clinical trial for the local administration of fluorouracil in CRC using colon hydrogel is associated with the registration number NCT06385418; the clinical trial comparing ADM hydrogel and alginate dressings in the efficacy of chronic wound treatment is associated with the registration number NCT06978569.

At present, the core scenarios of clinical exploration of PSHs in the field of CRC are mainly focused on postoperative recurrence prevention, intra-abdominal local administration, and sustained-release chemotherapy at tumor sites. However, such preparations still face challenges such as low mechanical strength and drug burst release during clinical transformation. In the future, they need to develop towards precision, combination, and integrated diagnosis and treatment, thereby promoting their transformation into a first-line treatment option for minimally invasive CRC therapy [117].

## 5. Safety Evaluation of PSHs

### 5.1. Safety of the Material Itself

The inherent safety of PSHs stems from the good biocompatibility, degradability, and low toxicity of natural polysaccharides such as chitosan and hyaluronic acid. Its degradability is the core of long-term safety, and the products after hydrolysis and enzymatic degradation are non-toxic and can be cleared by the body: the degradation products of chitosan-based hydrogels (0.4–0.7 kDa) are approximately 50% excreted through urine within 84 h without accumulation; the double-network hydrogels degrade after implantation in 2 weeks, matching tissue repair [121]; hydroxybutyl chitosan and hyaluronic acid-based hydrogels have no residues or accumulation risks after degradation. It should be noted that chemical modifications such as excessive acetylation may introduce safety risks.

### 5.2. Cell and Tissue Compatibility

PSHs exhibit good compatibility at the cellular, blood, and tissue levels. In vitro experiments (ISO 10993-5:2009 [122]) show that various hydrogels, such as those made from bee-derived chitosan, achieve a survival rate of >80% for cell lines like human corneal epithelial cells and a hemolysis rate of <5%, indicating no obvious cytotoxicity. In in vivo implantation experiments, hydrogels such as pectin/polyvinyl alcohol only cause mild inflammation after implantation, without tissue damage or other abnormalities [123,124]; there are no abnormal reactions at the immune level, and hyaluronic acid does not induce allergies.

### 5.3. Biological Safety in the Body

PSHs are generally considered safe and show no significant genetic toxicity under the tested conditions across various exposure stages in the body. In acute toxicity tests, hydrogels such as rooibos seed polysaccharide (LD_50_ > 9 g/kg for rats and rabbits) exhibited no obvious toxicity. In subacute toxicity tests, hydrogels such as oxidized dextran-based hydrogels resulted in normal physiological indicators after implantation. In chronic toxicity tests, oral administration of chitosan for one month did not adversely affect liver and kidney functions in mice. Genetic toxicity tests were negative under the conditions tested, with no observed mutagenicity [123].

### 5.4. Safety Related to Administration Routes

In different administration scenarios, PSHs balance safety and function. In CRC treatment, carboxymethyl cellulose hydrogels can locally release drugs and reduce systemic toxicity, and TME-responsive hydrogels can target drug release; in clinical use (primarily by injection), hyaluronic acid has been free of significant adverse reactions; in animal studies, it showed no toxicity, immunogenicity, sensitization, reproductive/developmental toxicity, or genotoxicity [125].

### 5.5. Evaluation of the Long-Term Safety of PSHs

Although PSHs hold great promise for CRC diagnosis and treatment, their long-term safety requires thorough assessment. The following five aspects merit particular attention.

Long-term toxicity: While the polysaccharide matrix is generally biocompatible, subchronic systemic toxicity should be evaluated in animal models for at least 12 weeks following hydrogel retention in the colorectal lumen or repeated administration. Existing studies report no oral, dermal, or ocular toxicity in acute tests of chitosan/fenugreek-g-poly(methylacrylate) hydrogels [126]; similarly, capecitabine-loaded chitosan/agarose-g-poly(methylacrylate) hydrogels showed no toxicity [127].

Residual crosslinking agents: Incompletely removed chemical crosslinkers may cause DNA damage and cytotoxicity in colonic mucosa. Pereira et al. evaluated oxidized dextran-based hydrogels using comet, micronucleus, and Ames tests; no significant DNA or chromosomal damage was observed at non-cytotoxic concentrations (≤3.5 mg/mL) [128]. Qiu et al. found that BDDE-crosslinked Bletilla striata PSHs exhibited good biocompatibility and degradability in cytotoxicity, hemolysis, and degradation assays [129].

Chronic inflammation: Physical barriers or degraded particles from hydrogels may chronically stimulate gut-associated lymphoid tissue. Notably, BDDE-crosslinked Bletilla striata PSHs promote M1-to-M2 macrophage polarization, alleviating inflammation during wound healing [129], offering insights for colorectal applications.

Microbiome alterations: Degradation products may shift gut microbiota composition. A fucoidan-based self-healing hydrogel (Fuco-PGAB) improved probiotic colonization, increasing survival by two orders of magnitude versus free strains, and regulated microbiota balance [130]. A Polygonum cuspidatum polysaccharide microgel specifically increased Akkermansia abundance, protecting the intestinal barrier [131].

Carcinogenic risk of degradation products: Aldehydes from oxidized polysaccharide degradation may cause oxidative DNA damage. Pereira et al. confirmed that oxidized dextran-based hydrogels and their metabolites showed no mutagenicity in Salmonella typhimurium and E. coli strains [128].

In summary, a multidimensional “degradation–toxicity–tumor microenvironment” evaluation system is recommended for future clinical translation studies to ensure the long-term safety of PSHs in CRC therapy.

## 6. Current Status and Future Prospects of Natural PSHs in the Diagnosis and Treatment of CRC

### 6.1. Current Challenges in the Use of Natural PSHs for the Diagnosis and Treatment of CRC

#### 6.1.1. Inherent Performance Limitations of the Materials

Stability and mechanical properties: Hydrogels formed by the physical crosslinking of natural polysaccharides alone have limited mechanical strength and are prone to rupture and loss of structural stability under the shear forces generated by intestinal peristalsis. Although mechanical properties can currently be enhanced through modification techniques such as polyelectrolyte complexation and dual-crosslinked networks, and factors like pH changes, enzymatic hydrolysis, and the TME can be converted into controllable responsive drug-release mechanisms using methods such as Schiff base crosslinking and tannic acid modification, the modification processes cannot fully offset the performance variations inherent to the raw materials [126]. Fluctuations in the natural properties of polysaccharides, such as molecular weight and deacetylation degree, lead to inconsistencies in the pore structure and crosslinking density of gels from different batches. Consequently, in vivo degradation and drug release behaviors vary, significantly affecting the stability of therapeutic efficacy [132]. Additionally, the in vivo degradation rate of these materials is difficult to precisely control; degradation products may disrupt the gut microbiota, and there is a potential risk of migration to distant organs. Large-scale production of complex modification systems is prone to performance degradation, and relevant long-term safety and quality standards remain to be established [133].

#### 6.1.2. Physiological and Microenvironmental Barriers to Colon-Targeted Delivery

Colon-targeted hydrogel delivery systems primarily face three major microenvironmental challenges. First is the mucus barrier: colonic mucus is dominated by MUC2 and consists of a dense inner layer and a loose outer layer. It is constantly renewed and readily clears exogenous carriers. Although existing modified hydrogels can enhance adhesion and retention, they struggle to maintain a dynamic balance between penetration and adhesion, and their targeting accuracy remains to be improved [134]. Second is interference from the gut microbiota: enzymes secreted by the microbiota degrade the hydrogel matrix. While this can be leveraged to achieve microbiota-responsive drug release, significant variations in the microbiota exist across individuals and pathological states. Combined with the influence of pathogenic bacteria, this leads to fluctuations in drug release behavior, creating an urgent need to establish standardized evaluation models [9]. Third, the in vivo pharmacokinetic research framework remains incomplete. Currently, research on targeted PSHs for CRC is largely confined to in vitro experiments, lacking systematic in vivo data on pharmacokinetic profiles, tissue distribution, metabolism, and accumulation. Studies by Koev et al. have demonstrated that starch-based colon delivery carriers can prolong drug retention time [135]; the potato starch–chitosan nanosystem developed by Throat et al. can enhance drug accumulation in the colon, reduce distribution to normal organs, and lower the toxicity of chemotherapy [136]. Currently, there is a lack of unified pharmacokinetic evaluation standards and detection methods for various PSHs, making it difficult to compare data across different studies. Future efforts should incorporate in vivo imaging and isotope tracing techniques into routine evaluations to establish a standardized in vivo pharmacokinetic research system. This will enable comprehensive analysis of the entire drug absorption, distribution, metabolism, and excretion chain, thereby providing data support for formulation optimization and clinical translation.

#### 6.1.3. The Gap Between Animal Models and Clinical Applications

Current mouse models differ significantly from humans in the following aspects, resulting in an extremely high risk of false positives:

At the genomic level: The somatic mutation profiles of AOM/DSS models are fundamentally different from those of human CRC [137].

Gut microbiome: The microbial communities of mice and humans differ markedly, affecting polysaccharide degradation and immune responses.

TME: Mouse tumors are small and have uniform blood supply, whereas human CRCs are often associated with a dense stroma, heterogeneous perfusion, and a metabolically repressed microenvironment. There is an urgent need to develop PDX models, genetically engineered models, humanized microbiome models, and organoid evaluation systems [138].

#### 6.1.4. Manufacturing Specifications

Raw materials: Fluctuations in molecular weight and degree of acetylation due to origin and harvest season [139].

Processing: Ion crosslinking is difficult to scale up linearly, and it is challenging to control the uniformity of microsphere particle size and encapsulation efficiency.

Sterilization: Wet heat sterilization causes chain degradation; ethylene oxide introduces toxic residues; γ-irradiation leads to chain breakage or excessive crosslinking. Electron beam irradiation of the lyophilized product is a viable approach.

Requirements: Establish a comprehensive GMP system covering raw material quality control, process validation, and sterilization validation [140].

#### 6.1.5. Regulatory and Approval Barriers

Multifunctional PSHs that combine drug delivery, imaging, and photothermal/immunotherapy present complex regulatory challenges. In China, sodium alginate hydrogels alone are typically regulated as Class III medical devices; however, once loaded with anticancer drugs, they are classified as drug-device combination products. The regulatory pathway must be determined based on their primary function, and such products are subject to joint review by the Drug Evaluation Center and the Medical Device Evaluation Center. According to the U.S. Federal Regulation 21 CFR 878.4018 [141], hydrophilic alginate dressings without added drugs are classified as Class I devices, whereas those containing drugs are excluded from this classification. This regulatory logic is consistent with the Chinese system, where the presence of drug components also triggers stricter classification requirements. If photothermal materials or contrast agents are further incorporated to form a diagnostic and therapeutic platform, the lack of clear classification guidelines may result in the application being accepted as either a medical device or an innovative drug. The preclinical evaluation requirements for these two categories differ significantly: medical devices emphasize biocompatibility and physical properties, while drugs require comprehensive pharmacokinetic and toxicological studies. Additionally, natural polysaccharide raw materials exhibit significant batch-to-batch variability and fluctuations in purity, often leading to insufficient process control. This necessitates multiple supplementary validation tests, significantly extending the development timeline and increasing costs.

#### 6.1.6. Patents and Industry Landscape

Strategy: Focused on ion-crosslinked microspheres, carbon material composites for reinforcement, and controlled release of specific drugs. Core applicants are primarily universities, with low industry participation.

Risks: Patents for basic crosslinking methods have expired, but functional modifications, nanomaterial composites, and combination therapy regimens remain protected. FTO analysis requires close attention.

Current Status: No products have been launched globally to date, reflecting a situation characterized by “active basic research but a low rate of commercialization” [142].

#### 6.1.7. Cost and Feasibility of Implementation

The clinical application of complex multifunctional hydrogels faces significant cost barriers. On the one hand, complex chemical modifications and nanomaterial composites significantly drive up raw material costs; on the other hand, reliance on high-end manufacturing processes—such as GMP production lines, microfluidic devices, irradiation, or aseptic techniques—further increases production costs. Taking CRC as an example, given the large patient population, if the cost of a single hydrogel treatment reaches several thousand yuan, it will be difficult to include it in the medical insurance system, severely limiting its clinical adoption. To address this issue, viable strategies include: streamlining non-essential functions to focus on core therapeutic needs (e.g., using simple physical crosslinking systems to replace complex chemical modifications) [143]; developing continuous production processes (e.g., continuous coating combined with UV curing) to replace batch-based, equipment-intensive production models; and reducing unit costs through large-scale production to ultimately achieve clinical accessibility of hydrogel formulations.

#### 6.1.8. Limitations of Existing Research

While there are significant differences in the triggering mechanisms of various polysaccharide systems (chitosan, pectin, alginate) for colon-targeted delivery, systematic comparisons under identical conditions remain limited [144]. The vast majority of studies only use a free drug as a control and have not yet conducted head-to-head comparisons with clinically approved formulations (such as 5-FU, capecitabine, and oxaliplatin) or mainstream delivery systems; no relevant clinical trials have been initiated globally, and a standardized evaluation framework is lacking [145]. Conclusions regarding drug synergistic effects vary across different polysaccharide carriers; the relationship between degradation and release remains controversial, with very limited supporting data currently available [146]. Unresolved technical challenges include: the absence of an IVIVC model, difficulties in programmably controlling degradation behavior, and a lack of long-term safety data. Regarding targeting mechanisms, the relative contributions of passive targeting (EPR effect) and active targeting are difficult to quantify [147]; the trade-off between ligand modification—sacrificing versatility for selectivity—remains unresolved.

### 6.2. Future Prospects for the Use of Natural PSHs in the Diagnosis and Treatment of CRC

#### 6.2.1. Upgraded Smart-Responsive Hydrogels

The complexity of the CRC microenvironment (pH gradients, elevated ROS, and GSH overexpression) necessitates multi-step “AND-gate” controlled release. Recent Advances include thiolated hyaluronic acid IPN hydrogel, which resulted in virtually no drug release in the stomach, sustained release in the colon, and 2.4-fold improved mucosal adhesion [148]. pH/ROS dual-responsive AuNP@MSN-chitosan hydrogel (2026) resulted in “On-demand unlocking” only at the tumor site [149]. Oxaliplatin-curcumin-Mn^2+^ coordination polymer hydrogel resulted in a segmented pH response, with in vivo drug release lasting >20 days [97]. Future Directions include higher-order response systems integrating multiple markers such as enzymes and ATP.

#### 6.2.2. Multifunctional Integrated Diagnostic and Therapeutic Platform

Closed-loop therapy: AIEgen-alginate hydrogel—in situ gelation in an acidic microenvironment to achieve photodynamic therapy + Ca^2+^ influx-induced mitochondrial damage + FLASH radiotherapy, combined to activate the immune system [150].

Postoperative Recurrence Prevention: Time-programmed laser-triggered immunogel—first releases curcumin to promote healing and then releases R848 to eliminate residual tumors, with a 40% response rate [151].

Trimodal Nanoplatform: Chemotherapy + photothermal therapy + radiotherapy integrated into an injectable adhesive hydrogel, achieving near-complete killing of HCT-116 cells [149].

#### 6.2.3. Gut Microbiome-Targeting Strategies

Chemotherapy Sensitization: Lactobacillus plantarum extracellular polysaccharides + cisplatin-alginate hydrogel—enriches SCFA-producing bacterial genera, enhances therapeutic efficacy, and reduces toxicity [152].

Immune Reprogramming: Synbiotic hydrogel capsule Lr@GI—Lactobacillus reuteri encapsulated in inulin/gelatin—depletes GSH, activates NLRP3, and induces M1 polarization [153].

Metabolic Reprogramming: Oral Lycium barbarum polysaccharide hydrogel—reprograms macrophage lipid metabolism via the TLR4-IRF3-LXRα axis to achieve chemotherapy sensitization and immune remodeling [100].

### 6.3. Optimization of Research on Natural PSHs for the Diagnosis and Treatment of CRC

The optimization directions for basic research are shown in Table 5.

Process and industrial optimization as shown in Table 6.

#### Regulatory, Patent, and Commercialization Strategy

According to the “Technical Guidelines for Clinical Trials of Drugs with Local Administration and Local Effects” (2022), inert carriers are developed under the modified new drug pathway and must provide local PK and safety data; active polysaccharides (such as Lycium polysaccharides) are developed under the innovative drug pathway and must provide complete pharmacodynamic and toxicological data, while establishing an internal quality control system in accordance with standards for polysaccharide biopharmaceuticals. Regarding patent strategy, conduct FTO analysis to identify potential patent barriers, identify gaps in the “carrier + active ingredient” dual-function synergy, and build a multi-tiered patent portfolio. Commercialization planning should monitor the latest patent trends in the field of oral macromolecular delivery from companies such as Gilead and BMS [154].

### 6.4. Comparison of Natural PSHs with Clinical Formulations and Mainstream Delivery Systems

The selection of comparison systems is shown in Table 7.

A quantitative comparison of key indicators is shown in Table 8.

#### Application Scenarios and Unique Characteristics of Each System

Chitosan-based: pH-responsive sol-gel transition + mucosal adhesion; suitable for in situ injection or scenarios requiring rapid retention after oral administration [155].

Pectin/alginate-based: Resistant to gastric acid and microbial hydrolysis; high targeting specificity; optimal for oral administration with colonic drug delivery. Mechanical strength requires optimization [156].

Composite systems (e.g., alginate/κ-carrageenan): Combine stability with immunomodulation (activation of NK cells), offering new insights for combined chemotherapy-immunotherapy [157].

## 7. Application of AI in Hydrogel Systems

**Figure 2:**
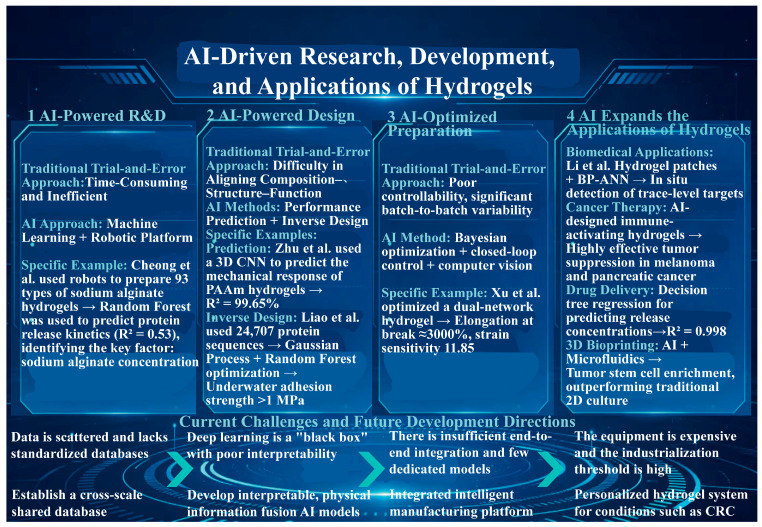
Schematic summary of AI-driven research, design, fabrication and biomedical applications of hydrogels together with existing challenges and future perspectives [159,160,161,162,163].

The deep integration of AI with materials science is bringing revolutionary changes to the research, design, preparation, and application of hydrogel systems. Especially in fields such as biomedical use, tumor treatment, drug delivery, and 3D bioprinting, AI, with its powerful data mining, nonlinear modeling, and autonomous optimization capabilities, has broken through the traditional bottlenecks of hydrogel research, such as trial-and-error reliance, long cycles, and poor controllability. It has promoted the transformation of hydrogel from “empirical design” to “precise manufacturing”, providing efficient solutions for the implementation of applications in multiple fields [158]. Examples of the research are shown in Table 9 and Figure 2.

### 7.1. AI Facilitating Hydrogel Research

Traditional hydrogel research relies on experimental trial and error, resulting in low efficiency and difficulty in exploring multidimensional formula spaces. AI technology uses machine learning algorithms to build data models, achieving intelligent upgrades in the research process, significantly shortening the cycle and improving accuracy. In data mining and optimization, AI can integrate multi-source experimental data to establish a correlation model between material composition, preparation conditions, and performance. For example, Cheong et al. used a robot platform to prepare 93 sodium alginate hydrogels and constructed a random forest regression model to predict the kinetics of protein drug release, clearly identifying that sodium alginate concentration was a key influencing factor [159]; Hashemi et al. utilized Bayesian optimization to optimize the composition of bio-ink, balancing printing performance and biocompatibility while regulating the hydrophobicity and degradation rate of the material [164].

In addition, AI can enable autonomous iterative optimization in research. Liao et al. [160] mined features from 24,707 adhesive protein sequences in the NCBI database and designed 180 biomimetic copolymer hydrogels. Through Gaussian process and random forest regression model iterations, they selected the optimal composition, enabling underwater adhesion strength to exceed 1 MPa, establishing a new paradigm of “protein sequence features—monomer composition—functional performance” correlation.

### 7.2. AI Empowering Hydrogel Design

The core of hydrogel design is to achieve precise matching of composition, structure, and function. Traditional methods are not sufficient for balancing multiple dimensions. AI, through nonlinear modeling and reverse design capabilities, achieves the leap from “performance prediction” to “directional design”. Performance prediction: AI models such as random forests and neural networks can accurately predict key performance indicators of hydrogels [165]. Zhu et al. [161] used PAAm hydrogels as the object and constructed 3D mesoscopic network models and 3D convolutional neural networks. After training with 2200 samples, the determination coefficient of the test set reached 99.65%, capable of accurately capturing the nonlinear mechanical response of heterogeneous networks; Cheong et al.’s random forest model had a cross-validation determination coefficient of 0.53 for the protein drug release kinetics of sodium alginate hydrogels, providing a basis for drug delivery adaptation design [159].

**Table 9:** Examples of AI-assisted hydrogel design and application research.

Applications	Authors (Year)	AI/Machine Learning Methods	Task	Key Results	Reference
Data Mining and R&D Acceleration	Cheong et al. (2026)	Random forest regression	Predict release kinetics of protein drugs from sodium alginate hydrogels	Identified sodium alginate concentration as the dominant influencing factor	[159]
	Hashemi et al. (2024)	Bayesian optimization	Optimize bioink composition to balance printability and biocompatibility	Realized tunable hydrophilic–hydrophobic properties and degradation rates	[164]
Performance Forecast	Zhu et al. (2021)	3D convolutional neural network (3D-CNN)	Predict nonlinear mechanical responses of heterogeneous polyacrylamide (PAAm) hydrogel networks	Coefficient of determination (R^2^) = 99.65% on test set	[161]
	Cheong et al. (2026)	Random forest regression	Predict protein drug release kinetics	Cross-validation R^2^ = 0.53	[159]
Reverse Engineering	Cadamuro et al. (2025)	Reverse prediction tool	Generate ECM-mimicking hydrogel formulations based on rheological properties	Enabled customized fabrication of colorectal cancer tissue engineering scaffolds	[166]
	Liao et al. (2025)	Gaussian process combined with random forest	Iterative optimization of biomimetic copolymer hydrogels based on 24,707 adhesive protein sequences	Underwater adhesion strength > 1 MPa	[160]
Optimization of Preparation Parameters	Xu et al. (2024)	Bayesian optimization coupled with random forest	Optimize concentrations of seven components in double-network hydrogels	Elongation at break ≈ 3000%, strain sensitivity = 11.85	[162]
	Mohammad et al. (2024)	Deep neural network (DNN)	Predict 3D bioprinting quality and optimize component ratios	Eliminated printing defects, including filament collapse and interlayer delamination	[167]
Process Monitoring and Closed-Loop Control	Sun et al. (2022)	Computer vision	Real-time monitoring of extrusion process and autonomous correction of printing defects	Significantly suppressed extrusion-based manufacturing defects	[168]
Biomedical Sensing	Li et al. (2026)	Back-propagation artificial neural network (BP-ANN)	In situ quantitative detection of trace analytes via hydrogel patches	Achieved in situ quantification of trace target substances	[163]
	Bilal et al. (2026)	Multimodal AI algorithm	Identification of MSI status and polyp risk stratification for colorectal cancer	Overall accuracy = 99.58%	[169]
Controlled-release and stimuli-responsive hydrogels for tumor treatment	Deshmukh et al. (2026)	LightGBM, XGBoost (gradient boosting); SHAP, LIME (explainability)	Predict drug release kinetics; optimize formulation parameters and crosslinking structures; enhance chemo-immunotherapy synergy; reshape TME	Superior predictive accuracy (R^2^ > 0.96) vs. classical kinetic models; enables AI-assisted design of immune-activating hydrogels with efficient tumor suppression in preclinical models	[170]
Drug Delivery	Schöning & Pfisterer (2023)	Decision tree regression	Simulate drug diffusion and release kinetics	Prediction R^2^ = 0.998	[171]
3D Bioprinting and Tumor Models	Yin et al. (2026)	AI integrated with microfluidics	Construct highly biomimetic tumor microenvironment models and enrich cancer stem cells	Superior drug screening performance compared with 2D cell culture	[172]

Reverse design: AI can automatically generate the optimal formula based on target performance. Cadamuro et al. [166] constructed a reverse prediction tool that can precisely output ECM simulation hydrogel formulas based on rheological properties, providing support for the customized preparation of CRC tissue engineering scaffolds; Bayesian optimization technology can quickly screen out light-degradable hydrogels with adjustable degradation rates and underwater adhesion hydrogels with high adhesion strength, achieving precise matching of function and formula.

### 7.3. AI Optimizing Hydrogel Preparation

Traditional hydrogel preparation has poor controllability and large batch differences, making it difficult to achieve large-scale precise preparation. AI uses real-time monitoring, parameter optimization, and closed-loop control to achieve intelligent regulation of the entire preparation process. Parameter optimization: Xu et al. optimized the concentrations of seven components for polyacrylamide/sodium alginate double-network hydrogels through Bayesian optimization and combined the random forest model to select the formula, forming a triple crosslinking structure, with the elongation rate of the hydrogel approaching 3000% and the strain sensitivity reaching 11.85 [162]; In 3D bioprinting, AI uses the DNN model to predict printing quality and optimize component ratios to solve defects such as filament collapse and inter-layer separation [167].

In terms of process monitoring and closed-loop control, AI, combined with technologies such as computer vision, continuously monitors the preparation process and autonomously corrects defects. Sun et al. developed a printing system integrated with computer vision, which can real-time regulate the extrusion motor to suppress defects [168]; the AI-driven closed-loop manufacturing system can achieve automated and high-throughput preparation of hydrogel scaffolds, control batch variations, meet GMP production requirements, and reduce production costs.

### 7.4. AI Expands Applications of Hydrogels

AI drives breakthrough applications of hydrogels in four core areas, accelerating clinical translation. In the biomedical field, Li et al. developed hydrogel patches combined with the BP-ANN model, which can achieve in situ quantitative detection of trace target substances [163]; implantable sensors combined with AI can non-invasively monitor physiological signals in the body, and the AI multimodal algorithm can accurately identify the MSI status and polyp risk stratification of CRC, reaching 99.58% [169]. AI can also optimize the parameters of 3D-printed scaffolds, providing support for postoperative tissue repair.

In the tumor treatment field, AI enables precise adaptation of hydrogels to the TME. Supervised machine learning algorithms, including LightGBM and XGBoost, have been systematically investigated for predictive modeling of drug release kinetics, demonstrating superior predictive accuracy (R^2^ > 0.96) compared to classical kinetic models, particularly for controlled-release and stimuli-responsive hydrogel systems. These algorithms, combined with feature engineering and explainability techniques such as SHAP and LIME, can optimize formulation parameters and crosslinking structures of intelligent responsive hydrogels, accurately predict drug release profiles, and enhance the synergistic effect of chemotherapy and immunotherapy. Furthermore, AI-assisted design of immune-activating hydrogels can optimize drug loading and release kinetics, reshape the TME, and achieve efficient tumor suppression in preclinical cancer models [170].

In the drug delivery and 3D bioprinting field, machine learning can simulate drug diffusion and release kinetics, and the decision tree regression model’s prediction coefficient for drug release concentration reaches 0.998, achieving precise control [171]; AI integrated with microfluidic technologies enables the construction of highly biomimetic tumor microenvironment models. Combined with 3D-bioprinted hydrogels, these systems support the enrichment of cancer stem cells and establish physiologically relevant in vitro platforms for drug screening, offering significantly improved accuracy and reliability over conventional 2D culture systems [172].

### 7.5. Existing Challenges and Future Development Directions

Although the application of AI in the hydrogel system has achieved remarkable results, it still faces four major challenges. The first is insufficient data quality and standardization, with scattered data and difficulty in multi-scale integration being an issue, as well as a lack of large-scale shared datasets, which restricts the generalization ability of the model. The second is weak model interpretability: the “black box” characteristic of deep learning makes it difficult to interpret the physical mechanism, affecting the credibility of clinical translation. The third is insufficient depth of technology integration, insufficient end-to-end integration, and scarcity of dedicated models. The fourth is high cost and high industrialization threshold, which is restricted by high-end equipment and algorithm applications and a lack of standardized processes, hindering large-scale implementation [165].

The future development direction is clear: First, build a cross-scale standardized database, integrate multi-dimensional and clinical data, and combine federated learning to ensure privacy. Second, develop physical information fusion models to enhance AI interpretability and achieve full-chain quantitative prediction. Third, promote integrated technology integration, develop high-throughput platforms and embedded control systems, and adapt to personalized cancer diagnosis and treatment. Fourth, focus on specific fields, develop customized hydrogels, and promote interdisciplinary collaborative innovation and clinical translation [173].

## 8. Conclusions

PSHs, with their excellent biocompatibility, degradability, and intelligent responsiveness, have effectively overcome the limitations of traditional CRC diagnosis and treatment, such as poor targeting, significant toxic and side effects, and low drug delivery efficiency, thus emerging as a research hotspot in the field of precise treatment for CRC. This review demonstrates that both single and composite PSHs can be fabricated into stable three-dimensional networks via various crosslinking approaches. Based on the diverse response mechanisms of the CRC TME, they can be combined with chemotherapy, immunotherapy, and other therapeutic modalities to achieve synergistic therapeutic effects, and their preclinical safety has been fully validated. However, these materials still encounter challenges, including insufficient mechanical stability, inadequate drug delivery performance, and difficulties in clinical translation. The integration of AI technology provides new pathways for the precise design, performance optimization, and large-scale production of hydrogels, which significantly enhances research efficiency. In the future, efforts should focus on material modification, multi-response synergy, and clinical translation. By combining with AI technology, it is necessary to break through existing bottlenecks, thereby promoting the translation of PSHs from basic research to clinical application and providing more efficient and safe novel strategies for CRC diagnosis and treatment.

## Figures and Tables

**Table 5:** Core optimization directions and task breakdown for fundamental research.

Direction	Specific Tasks
Improvement of in vivo evaluation	Systematically investigate ADME characteristics; conduct long-term toxicity assessment for no less than 6 months (intestinal barrier, gut microbiota and immune microenvironment)
Further optimization of animal models	Evolve from subcutaneous tumor models to PDX models, genetically engineered tumor models and chemically induced orthotopic tumor models, then to humanized microbiota models, and finally large animal models
Data standardization	Unify core parameters, including mechanical properties, degradation half-life and drug release profiles; establish an open-access database

**Table 6:** Core optimization directions and implementation tasks for the process and industrialization.

Direction	Specific Tasks
Scaled-up Preparation	Continuous-flow reaction & microfluidics; screen GMP-grade raw materials and establish quality control standards; explore high-value utilization of agricultural by-products
Sterilization & Storage	Compare sterile filtration, electron beam irradiation and supercritical CO_2_ sterilization; screen protective agents for lyophilized formulations; address stability challenges of wet dosage forms
Batch Variation Control	Implement Process Analytical Technology (PAT) to shift from end-point testing to in-process control

**Table 7:** Comparison of delivery systems for CRC.

Category	Representative Systems	Characteristics
Natural PSHs	Chitosan, Pectin, Alginate, κ-Carrageenan	Mucosal adhesion, enzymatic degradation by gut microbiota, immune regulation
Clinically approved drugs for CRC	5-FU, Oxaliplatin, Irinotecan	First-line agents with poor selectivity and severe toxicity
Mainstream commercial delivery systems	Eudragit coating, time-dependent pulsatile systems	Clinically applied; affected by individual differences in pH and gastrointestinal transit time

**Table 8:** Comparison of the performance of natural PSHs with conventional formulations/commercial systems.

Index	Conventional Preparations/Commercial Systems	PSHs
Drug loading capacity	Usually less than 10%	15%~25%
Colon-targeted drug release	Bioavailability < 20% with large individual differences	Sustained-release property; release profile adjustable via crosslinking degree
Degradation cycle	Poor degradability of synthetic coatings	24~72 h, matching the drug release profile
Tumor inhibition rate (mouse model)	30%~45% for free drug group	Significantly increased in drug-loaded PSH group. Existing studies have proven that natural PSHs can enhance the efficacy of chemotherapeutic drugs or inhibit tumors via immune regulation
Biocompatibility	Average performance of synthetic materials	Generally superior due to natural origin

## Data Availability

No new data were created or analyzed in this study. Data sharing is not applicable to this article.
